# The Impact of School Climate on Well-Being Experience and School Engagement: A Study With High-School Students

**DOI:** 10.3389/fpsyg.2019.02482

**Published:** 2019-11-05

**Authors:** Elisabetta Lombardi, Daniela Traficante, Roberta Bettoni, Ilaria Offredi, Marisa Giorgetti, Mirta Vernice

**Affiliations:** ^1^Department of Psychology, Catholic University of Milan, Milan, Italy; ^2^Carlo Besta Neurological Institute (IRCCS), Milan, Italy; ^3^Scientific Institute for Rehabilitation Medicine, Eugenio Medea (IRCCS), Bosisio Parini, Italy; ^4^Department of Psychology, University of Milano-Bicocca, Milan, Italy

**Keywords:** school climate, well-being, engagement, adolescents, personality traits

## Abstract

The aim of this work is to investigate the factors promoting students’ engagement at school and supporting their well-being experience. According to the Positive Education there is a strong relationship between school environment and student’s well-being. Moreover, the quality of the school climate perceived by the students was found to influence engagement in school activities, as well. In this study, 153 students (*M* = 67) attending 10th grade were presented with tests and questionnaires to assess individual assets (personality traits, literacy skills), emerging appraisals (school-climate, well-being experience) and emerging actions (school engagement), according to the *Student Well-Being Model.* Path analysis showed that the best model does include neither individual assets nor direct effect of school climate on engagement, as the effect of school climate on engagement is mediated by well-being experience. The main result is that school climate has been confirmed as an important factor to be considered to improve engagement in school activities, but it is effective only when its influence can modify the well-being experience of the students. Moreover, the lack of significant effects of individual assets in the model suggests that improving school climate means to support well-being experience and, indirectly school engagement, irrespective to learning abilities and personality traits. This work encourages working in/with schools to implement positive education programs that support and sustain a positive school climate and culture for school-community wellbeing.

## Introduction

In recent years, there is a growing interest in educational policies and research promoting student engagement at school in order to contrast the students’ passivity and the dropout rate (Archambault et al., 2009). As such, dropping out of high school has consequences for students’ well-being, including less lifetime earnings, more risky health behaviors, and poorer mental health (Archambault et al., 2009). In 2017, the dropout rate in Italy (13.8%) was higher than the EU average rate (10.7%) (source: MIUR, Italian Ministery of Education, University and Research, 2017), with more impact in the regions of the South of Italy. Furthermore, the percentage of 18–24 years old people who can be defined NEETs (Neither in Employment Nor in Education or Training) in 2017 was around 25.7% in Italy, a percentage which is nearly double the EU average percentage (14.3%) (source: European Union Commissione Europea, 2018).

In this scenario, research is needed to identify and support all factors that can reduce boredom and passiveness among young people. School enjoyment is influenced by different factors involving values, reading, and writing skills, expectations of social context (i.e., peers, teachers, and families) and is affected by both school and out-of-school contexts (Jennings, 2003; Ainley and Ainley, 2011). These aspects have been proved to affect learning outcomes and student’s engagement. The latter has been considered a key-factor to promote school completion and prevent dropout (Christenson and Thurlow, 2004; Ainley and Ainley, 2011; Christenson et al., 2012). Longitudinal studies showed that engagement in high-school is associated with educational and occupational outcomes in adulthood, as it not only predicts academic attainment, but also influences learner’s self-concept, along with adult educational and occupational achievement, irrespective from socioeconomic factors and personality traits (Abbott-Chapman et al., 2014). In this view, student’s engagement in school activities is a key protective factor against the risk of dropout (Finn, 2006; Archambault et al., 2009). Leaving school before completing high school education is often the outcome of problems that can be related to little support in school context or to health, personal, or emotional difficulties young people face. It can be also associated with socio-economic phenomena (i.e., the economic crisis), which have strong impact on family background (Berti et al., 2017). At the school level, a negative school climate (i.e., bullying or poor relationships between pupils and teachers) may trigger drop-out. Early school leaving, in addition, has significant societal and individual consequences, including the increased risk of unemployment, poverty, lower health, and social exclusion (Psacharopoulos and Patrinos, 2018). Data from 2012 indicated that in Europe 5.5 million of youth and young adults (18–24 years old) have not earned a high school diploma and were not currently enrolled in education and training (European Union Commissione Europea, 2013). In this scenario the study of the individual and contextual component affecting engagement in study activities can offer useful cues to face with huge social problems.

Engagement has been described in literature as a multidimensional construct, consisting mainly of three interrelated dimensions: emotion or affect, behavior, and cognition (Fredericks et al., 2004; Lam et al., 2014). The affective or emotional dimension of engagement refers to the young people’s attraction to school with the absence of negative emotions and the presence of positive emotions (i.e., interest, joy) during task involvement (Skinner et al., 2009). The behavioral aspect of engagement refers to factors (i.e., attention, effort, and persistence) that are in accordance with school expectations, learning-related tasks, and involvement in different school activities, even though not related to learning (Skinner et al., 2009). The cognitive face of engagement refers to the strategies used by the student in learning activity, the execution of a particular work style, and self-regulated learning (Fredericks et al., 2004; Wang et al., 2011). Very few studies have considered student engagement as a multi-dimensional construct. A recent large study by Fatou and Kubiszewski (2018), with high school students (enrolled in grades 10, 11, and 12), was aimed at examining possible associations between student engagement and school climate perceived by students. The main result of that work was that student engagement was associated with perceived school climate; more specifically, the researchers presented a model that explained a large proportion of the variance in students’ engagement by incorporating the perceived school climate. Such model was useful, in particular, for predicting affective engagement.

These findings support the idea that the school climate might play an important role to favor a positive school experience in students. Numerous approaches contribute to a conceptualization of school climate and there is not a unique definition of it. School climate is generally viewed as a multidimensional construct that encompasses a school’s atmosphere, culture, values, resources, and social networks (Wang and Degol, 2016). Furthermore, especially in the United States context, it is defined by the school norms, goals, values, interpersonal relationships, teaching and learning practices, organizational structures (National School Climate Council, 2007) and is studied in terms of school safety (e.g., anti-bullying). The U.S. Department of Education (2014), dispensed guidelines to promote and improve school climate and in 2018 the Office of Safe and Healthy Students proposed a compendium of school climate survey (American Institutes for Research, 2018). Several programs aimed at improving school climate have been developed to promote the quality of scholastic life (O’Brennan and Bradshaw, 2013). In fact, there is evidence that students are more engaged in school and attain higher academic achievement in schools with a positive school climate (Wang and Degol, 2016; Konold et al., 2018). School climate can be studied at the group level, by aggregating the data collection of the different actors (students, teachers, managers, parents) involved in the school context (Cornell et al., 2016). However, considering the perception of school climate also at an individual level can be very important, as several findings show that the feelings about school life have a great impact on student’s well-being (Gage et al., 2016).

School has been recognized as one of the most important developmental context, where students can acquire skills and competencies supporting their successful adaptation (Hamilton and Hamilton, 2009). However, there is still a limited perspective on factors that foster an optimal school environment (Norrish et al., 2013). These limits come from the prevalence of problem-focused approaches, instead of studies aimed to promote a positive educational context (Froh et al., 2011). In response to an excessive emphasis on research and practice related to weakness and disease, Positive Psychology movement redirected scientific inquiry toward the exploration of conditions promoting well-being in absence of pathology and illness (Seligman and Csikszentmihalyi, 2000; Snyder and McCullough, 2000; Sheldon and King, 2001; Rusk and Waters, 2013). Understanding factors associated with positive psychological experiences could provide meaningful guidance to plan interventions that improve the optimal functioning of children and young people at multiple levels.

The application of Positive Psychology in educational context gave rise to a new paradigm, the Positive Education. Seligman (2011) defined this approach as “traditional education focused on academic skill development, complemented by approaches that nurture wellbeing and promote good mental health” (p. 127). This conceptualization has implications for research, stressing the importance of the relationship between school environment and student health and well-being. “The fundamental goal of Positive Education is to promote flourishing or positive mental health within school community” (Norrish et al., 2013, p. 148). Seligman’s (2011) PERMA (Positive emotion, Engagement, Relationships, Meaning, and Accomplishments) model of flourishing claims that positive emotions, engagement, relationship, meaning, and accomplishment are the keys to happiness and well-being.

**FIGURE 1:**
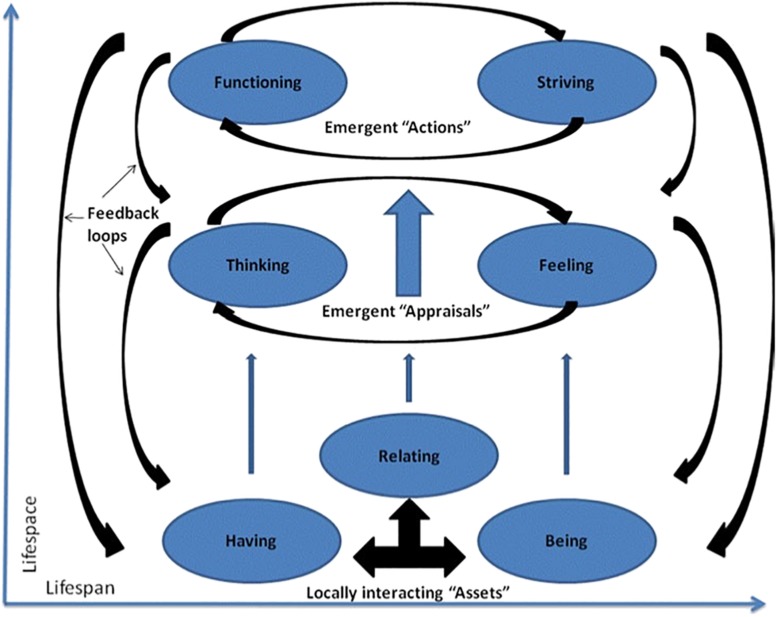
Student Well-Being Model (source: Soutter et al., 2014).

In this vein, Soutter et al. (2014) proposed a conceptual framework to investigate student well-being (the Student Well-Being Model: SWBM), in which seven domains are considered, and organized in three overarching categories (Figure 1): Having, Being, and Relating (Assets for well-being category); Feeling and Thinking (Appraisals category); Functioning and Striving (Actions category). The way these components interact is modeled according to the emergence mechanism: locally acting components give rise to higher-level entities (Roeser and Galloway, 2002), that interact with the other levels through feedback loops. In addition, the evolution of student well-being throughout the lifetime is also considered. It is worth noting that this model draws from Bronfenbrenner’s (1979) model of human development, as its components are considered embedded in the intersecting spheres of students’ lives, i.e., the classroom, school, family, community and natural and built environments. The aim of Soutter et al.’s (2014) work is to offer a framework for developing qualitative and quantitative measures of students’ well-being and for promoting well-being in school programs.

Among locally interacting assets, personality traits and attitude toward learning are expected to play a main role in school experience. Personality traits, described according to the so-called Five-Factors Approach (Block, 2010), are supposed to affect the way a student is used to face effort and duties (conscientiousness, neuroticism), to cope in front to new challenges (openness to experience), to interact with adults and peers (agreeableness, extraversion). Moreover, it is worth noting that students need to feel a close match between their current skills and abilities and instructional and curricular requirements, in order to have a positive school experience (Traficante et al., 2017). Due to the high social value attributed to literacy, students who are struggling in reading and writing often develop deep distrust in their own abilities, low motivation, helpless behavior, low self-esteem, and anxiety in being involved in school activities, as they anticipate their own failure (Morgan et al., 2008; Graham et al., 2012; Mason et al., 2012). Moreover, reading and writing abilities affect the social status of the child among the classmates (Elbaum et al., 1999; Cornoldi and De Beni, 2001; Mugnaini et al., 2009; Andolfi et al., 2015), with effects on school well-being. However, during the adolescence, students with learning disabilities might be able to apply compensative strategies that allow them to adequately deal with the school requests. Thanks to the support of the school context, the consequences of school experience difficulties might be reduced, the students can reach functional levels of learning and the real difficulties might occur only when they are under pressure (Fenzi and Cornoldi, 2015).

The aim of this work is to study, according to SWBM, how personal traits and literacy skills (locally interacting assets) influence students’ representations of school environment and of their experience of flourishing (emergent appraisals), and how these appraisals affect engagement in school activities (emergent actions). Fatou and Kubiszewski (2018) analyzed the effect of school-climate perception on engagement in high school, but in the present study other factors were considered as predictors of school engagement, and different models of relationships between different components were assessed. In particular, individual characteristics (personal traits and literacy skills) were expected to influence school-climate perception and well-being experience. Moreover, students’ well-being experience was supposed to influence engagement in school activities beyond the effect of school-climate perception, assessed by Fatou and Kubiszewski (2018).

## Materials and Methods

### Participants

One hundred fifty-nine (159, *M* = 15.6 years, *SD* = 6.2 months; Males = 70; 44%) high school students attending the 10th grade took part in the study. In this group there were 21 students with learning disabilities (13.2%), 2 students with sensory disabilities (1.2%), 4 students with other special needs (2.5%), and 28 students with Italian as their second language (17.6%). All students attended the 10th grade of three high-schools in the North of Italy during the 2018–2019 school year. Fifty-one participants were attending a technical institute (33.3%), 38 a vocational school (24.9%), and 61 a scientific high school (41.8%). Participants came from the middle class (*M* = 6.86, *SD* = 1.60), according to the Family Affluence Scale (FAS; Currie et al., 2008).

### Measures

#### Locally Interacting Assets

##### Literacy skills

1. Decoding ability is evaluated considering speed and accuracy in reading a list of pseudo-words (*DDE-2*, Sartori et al., 2007). Reading speed was measured both as the overall reading time (in seconds) and as the number of syllables per second. Reading accuracy was measured as the number of errors in reading aloud.
2. Reading comprehension was assessed through a standardized text reading test (*Advanced MT 2*, Cornoldi et al., 2010). Students were presented with 10 multiple-choice questions (four alternatives), after reading the text silently. The score was the number of correct answers (range 0–10).
3. Accuracy in spelling was assessed through a text dictation test (*Advanced MT 3*, Cornoldi et al., 2017). The experimenter dictated at a constant rhythm of one word every 2 s. The score was the number of incorrectly written words.

##### Personality traits

Italian adaptation of *Big Five Inventory* (BFI – John et al., 2008; It. ad. Ubbiali et al., 2013) was used to evaluate the personality traits. The questionnaire consists of 44 utterances referring to five trait dimensions of personality: extraversion (8 items, e.g., “*I am a person who*…generates a lot of enthusiasm”), agreeableness (9 items, e.g., “*I am a person who*…likes to cooperate with others”), conscientiousness (9 items, e.g., “*I am a person who*…makes plans and follows through with them”), neuroticism (8 items, e.g., “*I am a person who*…is depressed, blue”), and openness to experience (10 items, e.g., “*I am a person who*…is original, comes up with new ideas”). Answers were given on a 5-point Likert scale, from 1 = “strongly disagree” to 5 = “strongly agree.” Mean score for each dimension was carried out (range 1–5).

#### Emergent Appraisals

##### School climate

The *Georgia School Climate Survey* (GSCS) is annually administered as an anonymous survey in the Georgia, United States. The survey was developed by the Georgia Department of Education (GADOE) Assessment and Accountability Division, the Georgia Department of Public Health, and Georgia State University. The 20 items downloaded from the official website https://www.gadoe.org/Curriculum-Instruction-and-Assessment/Curriculum-and-Instruction/GSHS-II/Pages/Georgia-Student-Health-Survey-II.aspx cover the following areas: school connectedness, peer social support, adult social support, cultural acceptance, social/civic learning, physical environment, school safety, peer victimization, order and discipline, and parents’ involvement (e.g., “I feel connected to others at school”*;* “Teachers treat me with respect”; “My school building is well maintained”; “I feel safe in my school”). These items were administered after being translated in Italian and back-translated by an English native speaker. Answers were given on a 4-point Likert scale from 1 = “strongly disagree” to 4 = “strongly agree.” The overall school climate score ranged from 1 to 80.

##### Well-being experience

The *Comprehensive Inventory of Thriving* (CIT – Su et al., 2014) aims at assessing the general well-being through 54 items, pertaining to seven dimensions: (1) Relationships (6 scales, 18 items), composed by Support (e.g., “There are people who give me support and encouragement”), Community (e.g., I pitch in to help when my local community needs something done”), Respect (e.g., “People are polite to me”), Loneliness (e.g., “Often I feel left out”), Belonging (e.g., “I feel a sense of belonging in my Country”), and Trust (e.g., “Most people I meet are honest”); (2) Engagement (3 items: e.g., “I get fully absorbed in activities I do”); (3) Mastery (5 scales, 15 items), composed by Skills (e.g., “I use my skills a lot in my everyday life”), Learning (e.g., “I always learn something every day”), Accomplishment (e.g., “I am achieving most of my goals”), Self-Efficacy (e.g., “I believe that I am capable in most things”) and Self-worth (e.g., “The work I do is important for other people”); (4) Autonomy (3 items: e.g., “Other people decide most of my life decisions”); (5) Meaning (3 items: e.g., “I know what gives meaning to my life”); (6) Optimism (3 items: e.g., “I expect more good things in my life than bad”); (7) Subjective Well-being (3 scales, 9 items), composed by Life Satisfaction (e.g., “I am satisfied with my life”), Positive Feelings (e.g., “Most of the time, I feel happy”), and Negative Feelings (e.g., “Most of the time, I feel sad”). Items pertaining to the scales Loneliness, Autonomy, and Negative feelings were negatively phrased, so they were reversed. The rest of the items are phrased in a positive direction such that high scores mean that respondents view themselves positively in important areas of functioning. Participants were instructed to respond to each item on a scale from 1 = “strongly disagree” to 5 = “strongly agree.” Mean scores for each subscale were carried out (range: 1–5), and the CIT total score was the summed raw scores (range: 54–270).

#### Emergent Actions

Italian adaptation of the *Student Engagement Scale* (Lam et al., 2014; It. ad. Mameli and Passini, 2017), is a questionnaire that assesses the three dimensions of student engagement by three scales. The Affective engagement scale estimates students’ interests and positive inclination for learning and school (9 items: e.g., “I think what we are learning in school is interesting”); the Behavioral engagement scale investigates students’ involvement in school and extra-school activities and the effort in learning (12 items: e.g., “In class I work as hard as I can”). The Cognitive engagement scale measures students’ investment in learning processes and strategies (12 items: e.g., “Make up my own examples to help me understand the important concepts I learn from school”). In the first two scales (Affective and Behavioral engagement), students were asked to indicate their level of agreement on a 7-point Likert scale from 1 = “strongly disagree” to 7 = “strongly agree.” In the Cognitive engagement scale, students were asked to answer a 7-point Likert scale of frequency from 1 = “never” to 7 = “always.” The mean score for each subscale was carried out (range: 1–7).

### Procedures

After receiving the school-manager’s approval to carry out the research, the caregivers and the students were informed on the aim and procedure of the study. Parents provided a written consent for their children’s participation in the study and students gave informed written consent to the study, according to the *General Data Protection Regulation* (GDPR 2016/79, 25/05/2018). Students completed the questionnaires and the tests in two group sessions and their decoding ability was assessed in one individual session. The present study was approved by the Scientific and Ethics Committee of the Department of Psychology of Catholic University of Milan, in accordance with the Helsinki Declaration.

### Data Analysis

#### Normative Scoring

Standardized scores were computed from Italian normative data for literacy tests. Raw scores were recoded into *z*-scores, and the higher the value of *z*-scores is, the higher is the student’s ability.

#### Reliability Assessment

Reliability of each scale of the administered questionnaires was assessed through the Cronbach’s alpha, in order to include only reliable measures into the analyses.

#### Descriptive Statistics

Descriptive statistics were computed for each scale, in order to have a full description of the group of participants and verify the metric features of the variables included in the analyses.

#### Inferential Analyses

Canonical correlations (Pearson’s *r*) within all the measures were analyzed, in order to identify the relationships within all the variables of interest. Moreover, three linear regression analyses were carried out with engagement scales (Affective, Behavioral, Cognitive) as dependent variables one at a time, and four different set of independent variables: (a) personality traits (extraversion, agreeableness, conscientiousness, neuroticism, openness to experience), (b) literacy skills (decoding, comprehension, spelling), (c) well-being (total score), and (d) school climate (total score). Finally, path-analysis (SEM) was implemented by mean of Mplus 7.11 software (Muthén and Muthén, 1998-2015), to test the direct and indirect effects of individual assets, school climate and well-being on students’ engagement.

## Results

A score above 25th percentile rank at *Standard Progressive Matrices* test (SPM; Raven, 1954, 2008) was used as inclusion criterion, in order to obtain a good adherence to the tasks. The participants showing a SPM score above the 25th percentile was 153 (*M* = 15.6 years, *SD* = 6.5 months, Male = 67, 44%). In this group there were 18 students with learning disabilities (11.8%), 2 students with sensory disabilities (1.3%), and 3 students with other special needs (2%).

### Descriptive Statistics and Reliability Indexes

Descriptive statistics on the scores from the assessment of reading, writing and comprehension skills (Table 1) demonstrate the heterogeneity of the students considered in this study, as the minimum values show the presence of severe learning difficulties.

**TABLE 1:** Literacy measures: descriptive statistics of raw- and *z*-scores.

	**Raw scores**		***z*-scores**			
	***M***	***SD***	***M***	***SD***	**Min**	**Max**
Reading speed (syll/sec)	2.95	0.76	−0.17	0.93	−2.28	2.42
Reading accuracy (errors)	3.48	3.46	−0.23	0.54	−6.36	1.37
Spelling accuracy (errors)	4.23	4.22	0.33	0.90	−2.93	1.41
Comprehension (correct answers)	7.18	1.75	0.45	0.80	−2.38	1.75

As shown in Table 2, all the factors of *Big Five Inventory* show a good internal consistency: Cronbach’s alpha coefficients were adequate, as they ranged from 0.67 to 0.82. Also the subscales of *Comprehensive Inventory of Thriving* show a good internal consistency (from 0.60 to 0.88), as well as the *Georgia School Climate* (α = 0.80). Descriptive statistics and reliability indexes of the engagement scales used to assess the different dimensions of the students’ engagement show that all the scales of the *Student Engagement Scale* have a good internal consistency. Cronbach’s alpha coefficients were good as they ranged from 0.86 to 0.93.

**TABLE 2:** Personality traits, well-being, school-climate, and engagement: descriptive statistics and reliability indexes.

	**Scale**	***M***	***SD***	**Min**	**Max**	**Cronbach’s Alpha**
BFI	Extraversion	3.32	0.68	1.00	4.63	0.79
	Agreeableness	3.50	0.54	1.56	4.89	0.67
	Conscientious- ness	3.29	0.69	1.67	4.89	0.82
	Neuroticism	3.17	0.67	1.88	4.75	0.73
	Openness	3.33	0.62	2.00	4.90	0.74
GSCS	*Total Scale*	56.54	7.28	32	73	0.80
CIT	Relationships	3.41	0.49	2.06	4.50	0.83
	Engagement	3.53	0.64	1.00	5.00	0.60
	Mastery	3.46	0.51	1.80	4.53	0.87
	Autonomy	3.52	0.79	1.67	5.00	0.61
	Meaning	3.38	0.78	1.00	5.00	0.70
	Optimism	3.33	0.62	1.00	5.00	0.64
	Subjective well-being	3.51	0.69	1.00	5.00	0.88
	*Total Scale*	186.77	24.07	102	245	0.93
Engagement	Affective	4.48	0.95	1.44	6.78	0.86
	Behavioral	4.15	0.97	1.25	6.75	0.87
	Cognitive	4.36	1.1	1.17	7	0.93

### Correlation and Regression Analyses

Correlation analysis (Supplementary Table S1) was carried out to assess the associations between individual assets (personality traits, literacy skills), emergent appraisals (school climate, well-being experience), and emergent actions (engagement). First of all, it is worth noting that both comprehension and spelling accuracy are correlated with decoding ability, but are not related to each other. In other words, a good ability in transcoding graphemes-to-phonemes is associated both to a good text comprehension and to accuracy in spelling, but the latter two skills are not associated to each other. Moreover, good text comprehension is associated with high scores in Consciousness (*r* = 0.20) and Openness to experience (*r* = 0.172), with the perception of a positive school climate (*r* = 0.21) and with high level of engagement in learning activities (Affective: *r* = 0.179; Behavior: *r* = 0.169). Accuracy in spelling is associated with Neuroticism (*r* = 0.21): students who feel anxious and need to have a high level of control in their lives seem to be more accurate in spelling.

Overall, Supplementary Table S1 shows strong correlations among personality traits, perception of school climate and engagement in learning activities, in the expected direction. In order to disentangle the specific effects exerted by personality traits, literacy, well-being experience, and perception of the school climate on engagement in school activities, three multiple linear regression analyses (with backward method) were carried out on each of engagement dimensions.

#### Affective Engagement Scale

Table 3 shows the variables that contribute to the explanation of about 50% of the variance of the Affective engagement score (*R* = 0.71; *R*^2^ = 0.51; *F*_8_,_134_ = 17.44, *p* < 0.001). It is worth noting that only individual features and well-being experience seem to influence the affective engagement of students in school activities, whereas school climate has been excluded in previous steps. The personality profile of the student affectively involved in the learning process is characterized by conscientiousness, openness to experience and also by some degree of neuroticism. Students who are satisfied by their social relationships are usually engaged in their activities, and have an optimistic view of life and future. Their level of text comprehension is good. The negative coefficient of reading speed suggests that students who are attending 10th grade, in spite of their difficulties in reading, seem to be particularly engaged in learning activities.

**TABLE 3:** Affective engagement scale: linear regression coefficients.

	**Standardized coefficients B**	***t***	***p***
(Constant)		−2.428	0.016
BFI conscientiousness	0.223	3.034	0.003
BFI openness	0.162	2.308	0.023
BFI neuroticism	0.153	2.273	0.025
Reading comprehension	0.162	2.569	0.011
Reading speed	−0.186	−2.983	0.003
CIT Relationships	0.227	2.858	0.005
CIT engagement	0.246	2.825	0.005
CIT optimism	0.152	2.044	0.043

#### Behavioral Engagement Scale

Six variables contribute to the explanation of about 50% of the variance in Behavioral engagement score (*R* = 0.72; *R*^2^ = 0.52; *F*_6_,_136_ = 24.85, *p* < 0.001). Table 4 shows that students’ involvement in school and extra-school activities and the effort in learning are affected not only by individual features but also by perception of school climate. In other words, school context seems to influence the actual level of students’ participation to the school and extra-school activities. Students who are prone to being involved in school activity are characterized by conscientiousness, agreeableness, and attitude to be engaged, but seem to have low satisfaction in relationship. Also in this regression model the coefficient corresponding to reading speed is negative oriented, suggesting the students with less reading skills are more involved in school activities.

**TABLE 4:** Behavioral engagement scale: linear regression coefficients.

	**Standardized coefficients B**	***t***	***p***
(Constant)		−0.956	0.341
BFI conscientiousness	0.484	6.834	0.000
BFI agreeableness	0.177	2.551	0.012
Reading speed	−0.214	−3.481	0.001
School climate	0.222	2.738	0.007
CIT relationships	−0.249	−3.189	0.002
CIT engagement	0.221	2.797	0.006

#### Cognitive Engagement Scale

Only three variables were selected by the backward method (Table 5): openness to experience, reading speed, and sense of mastery. This model explained about 40% of the variance on Cognitive engagement score (*R* = 0.62; *R*^2^ = 0.39; *F*_3_,_139_ = 29.53, *p* < 0.001) and suggests that the application of metacognitive and strategic approach to learning activity is an attitude developed by students who are prone to face new experiences and feel a sense of mastery when faced with new challenges. This attitude seems to be less developed in students with lower level of reading skills.

**TABLE 5:** Cognitive engagement scale: linear regression coefficients.

	**Standardized coefficients B**	***t***	***p***
(Constant)		−2.051	0.042
BFI openness	0.305	4.421	0.000
Reading speed	−0.136	−2.029	0.044
CIT mastery	0.466	6.700	0.000

### Path Analysis

In order to draw a global representation of factors affecting engagement, structural equation a modeling technique was applied for the opportunity of testing and comparing different models of direct and indirect effects (Table 6).

**TABLE 6:** Path analysis: fit indexes of assessed models.

**Model**	**χ2**	***df***	**χ2*/df***	***CFI***	***TLI***	***RMSEA [90% CI]***	***SRMR***
1	352.06	115	3.06	0.714	0.662	0.117 [0.103–0.131]	0.113
2	81.06	33	2.45	0.904	0.869	0.099 [0.072–0.126]	0.066
3	81.01	34	2.38	0.906	0.876	0.096 [0.069–0.123]	0.066

**FIGURE 2:**
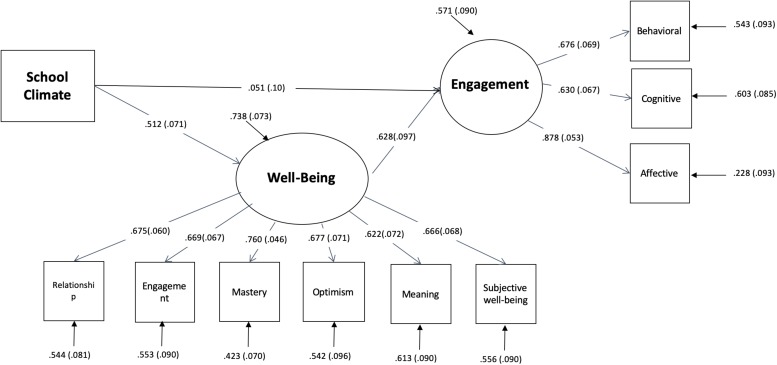
Path analysis: Model 2.

Fit indexes of Model 1, including individual assets (the latent variables “literacy skills” and “personality traits”) as independent variables, emergent appraisals (the latent variable “well-being” and the observed variable “school-climate”) as mediating variables, and emergent actions (the latent variable “school engagement”) as outcome were not satisfactory, due to the low impact of individual assets on school-climate appraisal and of literacy skills on well-being. For this reason, individual assets were excluded from the analyses and two different models were tested. In Model 2 (Figure 2) the direct impact of school climate on student’s engagement was tested, according to Fatou and Kubiszewski (2018) work, but also the direct impact of school climate on well-being and of well-being on engagement were assessed, due to the stress of Positive Education on the effect of school community on well-being (Seligman, 2011). Fit indexes improved a lot in comparison to the previous model, but the direct effect of school climate on engagement was far from significance level.

**FIGURE 3:**
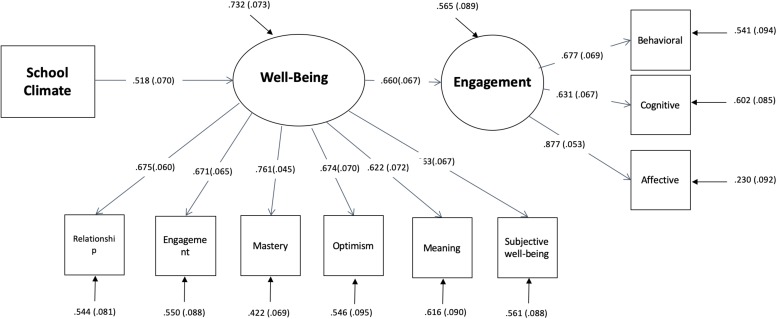
Path analysis: Model 3.

So, in Model 3 (Figure 3), such a direct effect was deleted and the impact of school climate on engagement was modeled as fully mediated by the impact that school climate exert on well-being experience. All the effects in Model 3 are highly significant, so it has been considered the best model.

## Discussion

The aim of this work is to assess the relationships within the components proposed by the SWBM by Soutter et al. (2014) in the experience of students attending 10th grade, in order to identify the aspects which should become the targets of interventions, planned according to the Positive Education approach. Recently, Fatou and Kubiszewski (2018) found that the quality of the school climate perceived by the students explains a high proportion of variance in the level of engagement in school activities, showing a direct impact of school environment on the interest the students develop in learning and in participating to educational proposals. In the present work this model was extended through the inclusion of other variables suggested by SWB model. In particular, the impacts of personality traits and literacy skills (assets) on school climate and well-being experience (appraisals), and the effects of appraisal components on engagement in school activities (actions) were assessed. Our results support our hypotheses, showing an impact of assets and appraisals on the student actions and revealing that well-being experience influence engagement in school activities beyond the effect of school-climate perception.

Correlational analyses showed that higher ability in text comprehension is associated to consciousness, openness to experience, perception of positive school climate, and high level of affective and behavioral engagement. The association between ability in text comprehension and deep interest in knowledge and in cultural experience suggests that students with high level of openness to experience, and attitude to acquire new information are more likely to develop reading habit. Moreover, both text comprehension and social and emotional competence, which can contribute to a positive appraisal of school environment and activities, require inferential skills and an attitude to assume different points of view. According to research demonstrating the relationship between social competence, perceived social support and engagement (e.g., Estell and Perdue, 2013), these results show that the characteristics of personality that underline social functioning are associated with positive representation of school climate. Furthermore, the associations of personal traits with the affective and behavioral engagement are relevant because it suggests that consciousness and openness to experience are related with cognitive and emotional involvement in study activities.

It is worth noting also the lack of significant correlation between literacy and well-being. Previous work (see Traficante et al., 2017) suggested that well-being experience of primary-school children is mainly affected by literacy skills, as education, in low grades, is focused on learning to read and to write. Differently from what has been found in primary school children, in 10th grade literacy skills seem not to influence students’ well-being anymore. Furthermore, this work suggests that students who are attending 10th grade, in spite of their difficulties in reading, seem to be particularly engaged in learning activities. This is in line with the evidence that high-school students with specific learning disabilities (SLD) can develop adaptive strategies to deal with the school requests and focus on functional level of learning (Fenzi and Cornoldi, 2015). Accordingly, a recent work on the students with SLD included in this sample, focused on the impact of low literacy skills on well-being experience (Sarti et al., 2019) did not found any significant difference between clinical and control groups. On the contrary, students with SLD showed an increasing sense of thriving related to a growing trust and perceived support from others.

The complex pattern of relationships within all the variables of interest was further analyzed through linear regression models. These models showed that affective engagement is affected by personality traits (consciousness, openness, and neuroticism) and literacy skills, as the higher the ability in text comprehension is, the more interested the student is in learning activities. However, it is worth noting that, consistently with the previous remark on students with learning disabilities, the lower the decoding skills are, the higher the affective engagement is in school. This unexpected result can be explained by taking into account that attending high school, in Italy, is not mandatory. So, if a student with learning disabilities chooses to study after finishing middle school, he/she must be very interested in learning activity. Moreover, regression coefficients show that students with a higher level of affective engagement are people with a positive attitude toward social relationships and have an active and positive representation of his/her life. Attitude to be engaged in school projects and extra-school educational activities (behavioral engagement) is predicted by traits concerning sociality (agreeableness, relationships) and involvement (conscientiousness, engagement) and is affected by school climate, as the higher the sense of belonging to the institution is, the higher the behavioral engagement. Again, students with lower decoding skills seem to be more active in their school, and are more prone to apply cognitive strategies in school activities (cognitive engagement). Such metacognitive attitude is also predicted by the sense of mastery and by openness to experiences.

Finally, path analyses allowed to disentangle this complex pattern of reciprocal relationships, through the assessment of different models, in which, according to SWBM (Soutter et al., 2014), individual assets (personality traits, literacy skills) were considered independent variables affecting appraisals (school-climate, well-being experience) and actions (school engagement). Results showed that the best model includes neither individual assets nor direct effect of school climate on engagement, which was suggested by Fatou and Kubiszewski (2018). The effect of school climate on engagement is mediated by well-being experience. In other words, school climate has been confirmed as an important factor to be considered to improve engagement in school activities, but it is effective only when its influence can modify the well-being experience of the students.

These results support the perspective of Positive Education, as intervention on school environment is expected to exert positive effects not only on students’ well-being, but also on their engagement in school activities and learning, irrespective to students’ assets. This work encourages working in/with schools to implement positive education programs that support and sustain a positive school climate and culture for school-community wellbeing.

## Data Availability Statement

The datasets generated for this study are available on request to the corresponding author.

## Ethics Statement

The studies involving human participants were reviewed and approved by the Scientific and Ethics Committee of the Department of Psychology of Catholic University of Milan. Written informed consent to participate in this study was provided by the participants’ legal guardian/next of kin.

## Author Contributions

EL and DT carried out data analyses and wrote the manuscript. All the authors contributed to data collection, the discussion of the results, and the planning and discussion of the draft.

## Conflict of Interest

The authors declare that the research was conducted in the absence of any commercial or financial relationships that could be construed as a potential conflict of interest.
