# Supplementary material for: The Impact of School Climate on Well-Being Experience and School Engagement: A Study With High-School Students
Source: Front Psychol. 2019 Nov 5;10:2482. doi: 10.3389/fpsyg.2019.02482 (PMC6848455; doi:10.3389/fpsyg.2019.02482)
Supplement: Supplementary file 1 [file Table_1.docx]

**Table S1** Correlations between personality traits, well-being experience and engagement

|  | Measures | Literacy | | | | Personality traits | | | | | GSCS | Well-being | | | | | | | | Engagement | |
| --- | --- | --- | --- | --- | --- | --- | --- | --- | --- | --- | --- | --- | --- | --- | --- | --- | --- | --- | --- | --- | --- |
|  |  | 1 | 2 | 3 | 4 | 5 | 6 | 7 | 8 | 9 | 10 | 11 | 12 | 13 | 14 | 15 | 16 | 17 | 18 | 19 | 20 |
| 1 | Reading speed |  |  |  |  |  |  |  |  |  |  |  |  |  |  |  |  |  |  |  |  |
| 2 | Reading accuracy | .374** |  |  |  |  |  |  |  |  |  |  |  |  |  |  |  |  |  |  |  |
| 3 | Comprehension | .181* | .250** |  |  |  |  |  |  |  |  |  |  |  |  |  |  |  |  |  |  |
| 4 | Spelling accuracy | .348** | .317** | 0.085 |  |  |  |  |  |  |  |  |  |  |  |  |  |  |  |  |  |
| 5 | BFI_Extraversion | 0.079 | 0.035 | 0.001 | -0.031 |  |  |  |  |  |  |  |  |  |  |  |  |  |  |  |  |
| 6 | BFI_Agreeabl. | 0.014 | -0.041 | 0.030 | -0.004 | .165* |  |  |  |  |  |  |  |  |  |  |  |  |  |  |  |
| 7 | BFI_Conscient. | .179* | -0.001 | .200* | 0.092 | 0.115 | .291** |  |  |  |  |  |  |  |  |  |  |  |  |  |  |
| 8 | BFI_Nevroticism | -0.047 | 0.055 | -0.014 | .209* | -.254** | 0.022 | -.162* |  |  |  |  |  |  |  |  |  |  |  |  |  |
| 9 | BFI_Openness | 0.053 | 0.056 | .172* | 0.023 | 0.157 | .198* | .228** | 0.077 |  |  |  |  |  |  |  |  |  |  |  |  |
| 10 | Schoool climate | .192* | .178* | .206* | -0.059 | .302** | .441** | .320** | -.164* | .269** |  |  |  |  |  |  |  |  |  |  |  |
| 11 | CIT_Relationships | 0.057 | 0.030 | -0.022 | -0.113 | .380** | .411** | .295** | -.326** | -0.054 | .594** |  |  |  |  |  |  |  |  |  |  |
| 12 | CIT_Engagement | 0.121 | -0.003 | 0.036 | 0.002 | .330** | .357** | .516** | -0.127 | .385** | .492** | .507** |  |  |  |  |  |  |  |  |  |
| 13 | CIT_Mastery | 0.139 | 0.010 | 0.017 | 0.075 | .276** | .328** | .562** | -.185* | .272** | .496** | .583** | .749** |  |  |  |  |  |  |  |  |
| 14 | CIT_Autonomy | 0.111 | -0.049 | -0.091 | -0.059 | 0.085 | -0.015 | 0.143 | -0.115 | .197* | 0.052 | 0.033 | 0.150 | .179* |  |  |  |  |  |  |  |
| 15 | CIT_Meaning | 0.075 | -0.039 | -0.035 | -0.021 | .207* | .264** | .400** | -.300** | 0.128 | .321** | .407** | .510** | .647** | 0.081 |  |  |  |  |  |  |
| 16 | CIT_Optimism | 0.066 | -0.044 | -0.004 | -0.127 | .344** | .285** | .319** | -.384** | 0.155 | .354** | .468** | .427** | .552** | 0.093 | .580** |  |  |  |  |  |
| 17 | CIT_SW | 0.129 | 0.056 | -0.054 | -0.045 | .453** | .246** | .206* | -.517** | -0.048 | .417** | .622** | .429** | .526** | 0.151 | .507** | .651** |  |  |  |  |
| 18 | CIT_total score | 0.130 | 0.014 | -0.029 | -0.067 | .442** | .396** | .458** | -.420** | 0.128 | .590** | .839** | .723** | .833** | .243** | .679** | .709** | .820** |  |  |  |
| 19 | Eng_Affective | -0.073 | -0.085 | .179* | -0.115 | .269** | .359** | .467** | -0.042 | .340** | .480** | .427** | .549** | .518** | -0.030 | .343** | .361** | .299** | .504** |  |  |
| 20 | Eng_Behavior | -0.072 | -0.049 | .169* | 0.047 | 0.034 | .379** | .591** | -0.001 | .319** | .382** | .206* | .483** | .491** | -0.001 | .315** | .221** | 0.111 | .338** | .663** |  |
| 21 | Eng_Cognitive | -0.079 | -0.154 | 0.107 | -0.022 | 0.127 | .265** | .423** | -0.080 | .442** | .276** | .193* | .470** | .488** | 0.121 | .342** | .235** | 0.130 | .355** | .546** | .535** |

* p < .05

** p < .01
